# Combining Ability Analysis in Complete Diallel Cross of Watermelon (*Citrullus lanatus* (Thunb.) Matsum. & Nakai)

**DOI:** 10.1100/2012/543158

**Published:** 2012-04-01

**Authors:** M. Bahari, M. Y. Rafii, G. B. Saleh, M. A. Latif

**Affiliations:** ^1^Malaysian Agricultural Research and Development Institute, Bukit Tangga, 06050 Bukit Kayu Hitam, Kedah, Malaysia; ^2^Department of Crop Science, Faculty of Agriculture, Universiti Putra Malaysia, 43400 UPM Serdang, Selangor, Malaysia; ^3^Institute of Tropical Agriculture, Universiti Putra Malaysia, 43400 UPM Serdang, Selangor, Malaysia; ^4^Plant Pathology Division, Bangladesh Rice Research Institute (BRRI), Gazipur-1701, Bangladesh

## Abstract

The experiments were carried out in two research stations (MARDI Bukit Tangga, Kedah, and MARDI Seberang Perai, Penang) in Malaysia. The crossings were performed using the four inbred lines in complete diallel cross including selfs and reciprocals. We evaluated the yield components and fruit characters such as fruit yield per plant, vine length, days to fruit maturity, fruit weight, total soluble solid content, and rind thickness over a period of two planting seasons. General combining ability and its interaction with locations were statistically significant for all characteristics except number of fruits per plant across the environments. Results indicated that the additive genetic effects were important to the inheritance of these traits and the expression of additive genes was influenced greatly by environments. In addition, specific combining ability effect was statistically evident for fruit yield per plant, vine length, days to first female flower, and fruit weight. Most of the characters are simultaneously controlled by additive and nonadditive gene effects. This study demonstrated that the highest potential and promising among the crosses was cross P2 (BL-14) × P3 (6372-4), which possessed prolific plants, with early maturity, medium fruit weight and high soluble solid contents. Therefore this hybrid might be utilized for developing high yielding watermelon cultivars and may be recommended for commercial cultivation.

## 1. Introduction

Watermelon (*Citrullus lanatus* (Thunb.) Matsum. & Nakai) is a very popular short-term nonseasonal fruit in Malaysia and has been classified under major fruits by the Ministry of Agriculture and Agro-Based Industry. Its refreshing and diuretic properties, associated with the pleasant taste and being a low-calorie fruit, make it a great alternative for the most varied diets. Malaysia occupied the 41st place in all watermelon-producing countries, with a production of 154,416 tones and a harvested area of approximately 9,241 hectares [1]. The main producing state is Johor, which contributes over 50% of national productions. At present, approximately 70% of productions are exported to Singapore, Taiwan, and Hong Kong. The world's largest producer of watermelon is China which usually accounts for over half of the world production [2].

Breeding for hybrid watermelon has so far achieved limited success in Malaysia. Several constraints in the breeding programme were faced, such as lack of genetic resources and crop failure attributed to high humidity, rainfall, and disease outbreaks [3]. Consequently there is limited choice of breeding material to work with and totally depends on commercial hybrids released by foreign seed companies. There is an ongoing need for watermelon improvement in country, especially for producing new hybrid cultivars with better fruit qualities comparable with the imported cultivars.

Estimating combining ability can be used to determine the usefulness of the inbred lines in hybrid combinations [4]. Selection of superior parents of hybridization is very important for watermelon improvement programmed, because the performance of a hybrid is related to the general (GCA) and specific (SCA) combining abilities of the inbred lines involved in the cross. According to Cruz and Vencovsky [5], the most promising hybrids are those that coming from the crossing of divergent parents, where at least one of them presented high GCA. Chaudhary [6] stated that diallel crosses are the most popular mating design and are used to obtain information on value of parents and to assess the gene action in various characters.

Some methods have been proposed to estimate the combining ability of genotypes to be used in breeding programs. Diallel analysis repeated over environments proposed by Matizinger et al. [7] is useful to estimate the combining ability of the parents. Information on the general and specific combining abilities and their interactions with environments will be helpful in the analysis and interpretation of the genetic basis of important traits.

Several studies on GCA and SCA in watermelon had been done by many researchers such as Brar and Sukhija [8], Brar and Nandpuri [9], and Sidhu and Brar [10]. In another study, Souza et al. [11] worked on 3 × 3 diallel analysis in watermelon and reported that most of the characters studied are simultaneously controlled by additive and nonadditive gene effects. Their work demonstrated that the potential of the cross is between “Sugar Baby” and “Kodama” which had prolific plants, small fruit and high soluble solids content. While Ferreira et al. [12] reported that GCA effects were more important than SCA effects for number and weight of fruit per plant, as well as for flesh colour, thickness, and soluble solid content, for number of days to the first female flower and number of seeds, indicating the predominance of non-additive gene effects was found.

The main aim of the research was to identify breeding lines having good ability effects for yield components and fruit characters. Hence, the present study was to estimate  the combining ability of four watermelon inbred lines and identify the important characters to support the breeding program of watermelon in the country.

## 2. Material and Methods

The materials for this study consisted of four watermelon inbred lines and their F_1_ hybrids including the reciprocals. The four inbred lines used as parents were CS-19-S_7_ (P1), BL-14-S_7 _ (P2), 6372-4-S_7_ (P3), and CH-8-S_7_ (P4). Four parents were crossed in complete diallel mating scheme in greenhouse at Bukit Tangga MARDI station in the second half of 2008. Two varieties, C1 and C2, were used as check.

The 18 genotypes (4 parents, 6 F_1 _ hybrids (P1 × P2, P1 × P3, P1 × P4, P2 × P3, P2 × P4, and P3 × P4), 6 F_1_ reciprocal hybrids (P2 × P1, P3 × P1, P4 × P1, P3 × P2, P4 × P2, and P4 × P3), and two check varieties) were evaluated in a randomized block design with 4 replications at Bukit Tangga MARDI Research Station and Seberang Prai MARDI Research Station, over a period of two planting seasons. Each plot consisted of 7 plants spaced 1.2 m apart in rows and 2.5 m between rows. Details of two environments are presented in Table 1.

The seeding was carried out in trays, and the seedlings were transplanted 14 days after sowing. Fertilization was performed by applying, in foundation, the rates of 500 kg/ha of NPK Green. Cultural practices were conducted according to technical recommendations for the culture in the country. The identification of ripe fruit was based on observation of drying of the tendril adjacent to the stalk and the sound emitted by woody fruit when struck by his fingertips. Plants were evaluated on the yield components (fruit yield, number of fruits per plant, vine length, number of days for the appearance of the first female flowers, and days to fruit maturity) and fruit characters (fruit weight, total soluble solids and rind thickness). To obtain the means of the variables in each plot, we sampled five plants and five fruits, taken at random during the harvest.

 Estimates of the general and specific combining ability effects were obtained using the methodology proposed by Griffing [13] for analysis of diallel with parents, F_1_ and F_1_ reciprocal (Method I), considering fixed effect of treatments. The analysis of variance of diallel was performed according to the scheme presented by Zhang and Kang [14]. This program is considered as an effective method in analyzing and interpreting diallel cross data which are conducted in a number of environments [15].

## 3. Results

### 3.1. Combined ANOVA

A combined analysis of variance was performed on the data of yield components and fruit traits to estimate the amount of variability for these characteristics among parents, their F_1_ and F_1_ reciprocals. Analysis of variance for diallel cross of watermelon genotypes over the four environments is presented in Table 2. Results showed that significant differences were found among the environments (E) and among the genotypes (parents and off springs) for the yield components (fruit yield, number of fruits, vine length, days to the first female flowers, and days to fruit maturity) and fruit characters (fruit weight, total soluble solids, and rind thickness). Mean data for yield components and fruit characters are given in Table 3.

Analysis of variance of combining ability indicated that effects of general combining ability (GCA) were found to be significant for all yield components and fruit characters except number of fruits (Table 2). However the interaction of GCA effects and environment (GCA × E) was statistically evident for vine length, days to first female flower, days to fruit maturity, fruit weight, total soluble solids, and rind thickness.

For specific combining ability (SCA) analysis, significant effects were found for all traits measured except number of fruits and total soluble solids (Table 2). However the interaction of SCA effects and environment (SCA × E) was significantly differe for vine length, days to first female flower and days to fruit maturity. Analysis of variance for reciprocal effects (R) showed that significant effects were found for fruit yield, number of fruits, and days to maturity. Meanwhile, the interaction of reciprocal effects and environment (R × E) was nonsignificant for all traits measured except days to maturity and total soluble solid content (Table 2).

### 3.2. Analysis of Combining Ability

#### 3.2.1. Yield Components

The parent with the highest ranking for fruit yield was P1 (14.8 kg). This parent also showed significant positive GCA effects across environment (1.41) for fruit yield. Among the F_1_ hybrids, P3 × P2 (15.19 kg), followed by P2 × P3 (14.98 kg), displayed the best performance for this character (Table 3). The estimated specific combining ability (SCA) effects revealed that hybrid P2 × P3 gave significant positive SCA effects of 1.59 for fruit yield per plant across the environments.   It had the higher positive reciprocal effects values for number of fruit, days to female flower, total soluble solids, and rind thickness (Table 5).

With regards to number of fruits per plant, the check variety C2 (3.6 fruits) was significantly higher than all genotypes (Table 3). However, the parent with the highest ranking for number of fruits was P1 (2.94), which had the highest positive and significant GCA value of 0.11 (Table 4). Among the F_1_ hybrids, the highest number of fruits was recorded at P3 × P2 (2.9 fruits), followed by P3 × P1 (2.8 fruits) and P4 × P1 (2.7 fruits). The highest SCA positive value for number of fruits was found at P2 × P3 (0.17). Meanwhile the highest positive reciprocal effect (R) value was observed in reciprocal hybrid, P4 × P3 (0.13) (Tables 3 and 5).

For vine length, parental line P1 (3.48 m) was significantly longer than all genotypes and had the highest positive GCA value of 0.27 (Tables 3 and 4). Of the hybrids, P2 × P1 (3.23 m) ranked the highest among the crosses for vine length (Table 3). However the estimated SCA effects showed that hybrid P2 × P3 had the highest positive SCA effects (0.13) for vine length (Table 5). Meanwhile the highest positive reciprocal effect (R) value was 0.04 and was observed in hybrid P2 × P1.

For the appearance of the first female flowers, the genotype with the earliest flowering was P2 (BL-14) (31.1 days) and the GCA effect was −0.64 (Tables 3 and 4). Among the hybrids, P1 × P4 was the earliest to the first female flower appearance and had the highest negative SCA effects (−1.04) (Table 5). The highest negative R effects for days to first female flowers across the environments were observed in cross P4 × P2 that was nonsignificant among the reciprocal hybrids.

Parental line P4 showed the earliest fruit maturity (67.1 days), which had the highest negative GCA effects (−1.34) (Tables 3 and 4). However, the estimated SCA effects showed that hybrid P2 × P3 had the highest negative SCA effects (−0.77) (Table 5). Meanwhile the highest negative R effects value was observed in hybrid P3 × P2 (−0.69).

#### 3.2.2. Fruit Characters

The parental line with the highest ranking for fruit weight was P1 (7.94 kg) which also gave significant positive GCA effects across the environments (0.886) (Tables 3 and 4). The highest SCA effect was measured at P1 × P4 (0.501), followed by P2 × P3 (0.463), which was significantly higher than that of the other crosses (Table 5).   However, the estimates of R effects for fruit weight across the environments showed that no hybrid revealed positive and significant R effects.

The result showed that check variety C2 was the highest ranking genotype for total soluble solids (11.16  ^0^Brix), and it was significantly different from other genotypes (Table 3). There was no parental inbred line which revealed significant and positive GCA effects for total soluble solids. However, inbred CH-8 (P4) gave the highest positive effect for total soluble solids (0.218) across the environments (Table 4). Estimates of SCA and R effects for total soluble solid content across the environments showed that no hybrid revealed positive and significant SCA effect (Table 5).

For rind thickness, inbred line CH-8 showed significant positive GCA effect for rind thickness (0.168) (Table 4). As a result, this parent contributed to produce hybrids with thick rind. The highest SCA effect was measured for P2 × P3 (0.11), which was significantly higher than that of other crosses (Table 5).

## 4. Discussion

The inbred and their hybrids were tested over different environments, and performance of all characters was investigated. These results indicated those environmental factors which varied from location to location and planting cycle to planting cycle and influenced the expression of the characteristics of watermelon parents and their progenies. Interaction of genotypes with environment (G × E) was found to be significant for the vine length, days to first female flower, days to fruit maturity, fruit weight, total soluble solids, and rind thickness, indicating that parents and their progenies were susceptible to environmental conditions. 

Parental line P1 was the highest fruit yielder, and this parent also showed significant positive GCA effects across the environment. This inbred line is a good combiner for fruit yield. In combining ability analysis, the effects of general combining ability (GCA) were found to be significant for all yield components and fruit characters except number of fruit yield. Results indicate that there is presence of additive and nonadditive gene actions. Mean squares were higher in general combining ability than that of mean squares of specific combining ability for majority traits. It is suggested that the additive gene effects are more important than the nonadditive gene effects. This finding is similar to that of the report of Souza et al. [11] as described in their study on three intercrossings of watermelon genotypes. The significant effect of GCA × E was found among the traits, vine length, days to first female flower, days to fruit maturity, fruit weight, total soluble solids, and rind thickness which revealed that the action of additive genes was influenced by the environmental variation. In addition, variation of these traits was influenced greatly by environments. 

Inbred P1 gave significant positive GCA effects across the environments for fruit yield, number of fruits, and vine length. Results suggested that this inbred contributed to achieving plants more compact, prolific, productive, and producing large fruit. Inbred P2 gave significant and that are highest negative values across environments for early in days to first female flower and early in days to maturity period, while inbred lines P3 and P4 showed significant positive effect across the environments for total soluble solids and rind thickness, respectively. It indicated that these parents contributed to producing hybrids with thick rind and sweeter fruits. The inbred lines that revealed strong GCA effects could be utilized further as sources for population improvement towards the accumulation of favorable additive genes in population for watermelon variety improvement in Malaysia. 

Significant SCA effects were found for most traits except number of fruits, total soluble solids, and rind thickness. Results from this study were not in agreement with Ferreira et al. [12] who reported that the number of fruits, total soluble solids, and rind thickness were significant in their study. So, our study revealed that hybrid P2 × P3 had the highest SCA effects for most of traits except days to flower.   It indicated that the significant role of nonadditive effects was involved in the inheritance of this trait. There was no hybrid which revealed significant and positive R effects for rind thickness, indicating that reciprocal effect is not important. 

Reciprocal effects (R) showed that significant effect was found for yield components, that is, fruit yield, number of fruit, and days to maturity. Similar results were reported by Feyzian et al. [16] and pointed out that reciprocal effects were significant for fruit yield and fruit maturity on melon.The interaction of reciprocal effects and environment (R × E) was nonsignificant for all traits measured except days to maturity and total soluble solids content. Therefore, extra chromosomal inheritance or maternal effects may be involved in gene control of these characters. The study of this type of gene interaction is important in breeding programs which allow the determination of parents to be used as donors or recipients of pollen. 

This study strongly suggests that those with strong SCA effects such as P2 × P3  could be advanced for hybrid variety release after other yield stability factors have been considered. 

## Figures and Tables

**Table 1:** Details of environmental conditions of two locations.

Descriptions	MARDI Seberang Prai (MSP)	MARDI Bukit Tangga (MBT)
Latitude	5°8′N	6°28′N
Longitude	100°32′E	100°32′E
Temperature	30°C	33°C
Annual rainfall	2670 mm	2480 mm
Humidity	90%	85%
Soil type	Sogo series	Kuah series

MARDI: Malaysian Agricultural Research Development Institute.

**Table 2:** Mean squares of combined ANOVA for yield components and fruit traits in diallel cross of four watermelon inbred lines over four environments.

Source of variation	d.f.	Fruit yield	Number of fruits	Vine length	Days to first female flower	Days to fruit maturity	Fruit weight	Total soluble solids	Rind thickness
Environments (E)	3	441.08**	6.42**	38.19**	579.05**	2162.11**	53.20**	116.21**	4.47**
Reps (environment)	12	15.99	0.45	0.18	2.76	8.00	1.44	0.47	0.15**
Genotypes (G)	15	46.15**	0.82*	1.34**	31.51**	113.10**	11.31**	1.49**	0.14**
GCA	3	114.40**	1.02	5.86**	92.09**	484.82**	46.26**	3.84**	0.20**
SCA	6	26.22*	0.38	0.36*	32.34**	31.21**	4.37**	0.93	0.21**
Reciprocal (R)	6	31.97**	1.15**	0.07	2.66	9.13*	0.77	0.87	0.04
G × E	45	15.55	0.45	0.29**	8.91**	37.23**	1.78*	0.95*	0.14**
GCA × E	9	16.37	0.24	0.55**	12.10**	77.13**	3.41**	1.29*	0.53**
SCA × E	18	14.62	0.63	0.28*	12.81**	42.42**	1.39	0.62	0.06
R × E	18	16.07	0.39	0.17	3.20	12.10*	1.36	1.11*	0.02
Error	180	10.04	0.39	0.15	3.24	6.76	1.24	0.65	0.039

^∗,  ∗∗^Significant at *P* ≤ 0.05 and *P* ≤ 0.01, respectively.

**Table 3:** Mean performance of parents and their hybrids for yield components and fruit traits in watermelon.

Genotype	Fruit yield (kg)	Number of fruits	Vine length (m)	Days to first female flower	Days to fruit maturity	Fruit weight (kg)	Total soluble solids (^0^Brix)	Rind thickness (cm)
P1 (CS-19)	14.80	2.94	3.48	37.38	77.75	7.94	8.91	1.53
P2 (BL-14)	10.31	2.25	2.60	31.06	67.56	5.20	9.26	1.34
P3 (6372-4)	10.76	2.38	2.76	31.55	71.13	5.47	9.02	1.31
P4 (CH-8)	11.77	2.56	2.42	31.75	67.06	5.56	9.65	1.50
P1 × P2	14.98	2.13	3.06	33.00	71.94	6.21	8.71	1.26
P1 × P3	11.97	2.56	3.04	33.82	72.94	6.42	8.82	1.25
P1 × P4	12.71	2.63	2.70	32.50	71.25	6.97	9.13	1.42
P2 × P3	14.98	2.81	2.98	31.94	71.88	6.20	9.07	1.29
P2 × P4	12.21	2.31	2.96	32.67	67.56	5.67	9.49	1.39
P3 × P4	10.69	2.44	2.61	32.06	70.38	5.22	9.49	1.33
P2 × P1	13.68	2.63	3.23	33.56	72.19	5.91	8.69	1.35
P3 × P1	13.66	2.88	2.92	32.63	68.94	5.80	9.11	1.44
P4 × P1	11.14	2.69	2.53	32.44	69.06	5.43	9.04	1.32
P3 × P2	15.19	2.90	2.72	32.63	72.19	7.48	9.67	1.55
P4 × P2	10.12	2.38	2.54	32.69	68.69	5.03	9.25	1.34
P4 × P3	11.10	2.44	2.56	33.06	69.88	5.44	9.05	1.30
C1 (check)	9.28	2.50	2.35	32.06	70.44	4.21	9.50	1.31
C2 (check)	8.11	3.63	2.63	31.88	67.56	2.51	11.16	1.70

Mean	11.88	2.59	2.78	32.69	70.47	5.70	9.28	1.40
CV (%)	28.17	24.30	21.00	6.76	5.03	20.45	9.59	18.58
LSD (5%)	1.53	0.44	0.27	1.22	2.47	0.81	0.62	0.32

**Table 4:** Estimation of general combining ability (GCA) of parental lines for characters measured in the F_1_ watermelon hybrids.

Character	CS-19 (P1)	BL-14 (P2)	6372-4 (P3)	CH-8 (P4)
Fruit yield	1.413**	−0.568*	−0.373	−0.472
Number of fruits	0.113*	−0.105*	−0.004	−0.004
Vine length	0.266**	−0.036	0.025	−0.255**
Days to first female flower	1.238**	−0.645**	−0.168	−0.426**
Days to fruit maturity	2.836**	−1.336**	−0.297	−1.203*
Fruit weight	0.886**	−0.431**	−0.294**	−0.161
Total soluble solids	−0.159**	0.058	−0.117	0.218
Rind thickness	−0.015	−0.073	−0.080	0.168**

^∗,  ∗∗^Significant at *P* ≤ 0.05 and *P* ≤ 0.01, respectively.

**Table 5:** Estimation of specific combining ability (SCA) and reciprocal (R) effects for characters measured in the F_1_ watermelon hybrids.

Hybrid	Fruit yield	Number of fruits	Vine length	Days to female flower	Days to maturity	Fruit weight	Total soluble solids	Rind thickness
P1 × P2	0.080	−0.074	−0.032	−0.910**	−0.242	−0.246	−0.157	−0.093
P1 × P3	−0.500	−0.051	0.022	−0.168	−0.625	−0.423*	−0.113	−0.062
P1 × P4	0.727	−0.051	−0.120*	−1.035**	−0.563	0.501*	0.192	0.052
P2 × P3	1.594**	0.168	0.128*	0.684**	−0.766	0.463*	0.210	0.110*
P2 × P4	−0.837	−0.020	0.050	0.660**	1.422	−0.280	−0.053	−0.044
P3 × P4	−0.321	0.035	−0.044	0.559*	0.320	−0.104	−0.205	−0.059
P2 × P1	−1.769**	−0.344**	0.040	0.531	0.031	0.006	−0.180	−0.018
P3 × P1	−0.856	−0.031	−0.094	0.125	0.375	0.253	0.064	−0.050
P4 × P1	−1.238	0.031	−0.008	−0.063	−0.469	−0.257	−0.273	−0.065
P3 × P2	−0.721	−0.281	0.020	0.031	−0.688	−0.068	0.191	−0.027
P4 × P2	0.285	0.031	0.034	−0.313	0.844	0.098	0.123	−0.003
P4 × P3	0.022	0.125	−0.013	−0.311	−0.406	−0.004	−0.006	0.012

^∗,  ∗∗^Significant at *P* ≤ 0.05 and *P* ≤ 0.01, respectively.
